# High BTLA Expression Likely Contributes to Contraction of the Regulatory T Cell Subset in Lupus Disease

**DOI:** 10.3389/fimmu.2021.767099

**Published:** 2021-11-25

**Authors:** Lucie Aubergeon, Matthieu Sawaf, Renaud Felten, Jacques-Eric Gottenberg, Hélène Dumortier, Fanny Monneaux

**Affiliations:** ^1^ CNRS UPR3572, Immunology, Immunopathology and Therapeutic Chemistry, Institute of Molecular and Cellular Biology, Strasbourg, France; ^2^ Rheumatology Department, National Reference Center for Autoimmune Diseases, Strasbourg University Hospital, Strasbourg, France

**Keywords:** systemic lupus erythematosus, BTLA, HVEM, regulatory T cells, inhibitory receptors

## Abstract

B and T lymphocyte attenuator (BTLA) is a co-inhibitory receptor that is expressed by lymphoid cells and regulates the immune response. Consistent with an inhibitory role for BTLA, the disease is exacerbated in BTLA-deficient lupus mice. We recently demonstrated that the BTLA pathway is altered in CD4^+^ T cells from lupus patients. In the present work, we aimed at delineating the expression pattern of BTLA on CD4^+^ T cell subsets suspected to play a key role in lupus pathogenesis, such as circulating follicular helper T cells (cT_FH_) and regulatory T cells (Tregs). We did not detect significant *ex vivo* variations of BTLA expression on total CD4^+^ T cells (naive and memory), cT_FH_ or T_FH_ subsets between lupus patients and healthy controls. However, we interestingly observed that BTLA expression is significantly increased on activated Tregs, but not resting Tregs, from lupus patients, especially those displaying an active disease. Moreover, it correlates with the diminution of the Tregs frequency observed in these patients. We also showed that both BTLA mRNA and protein expression remain low after TCR stimulation of activated Tregs sorted from healthy donors and evidenced a similar dynamic of BTLA and HVEM expression profile by human Tregs and effector CD4^+^ T cells upon T cell activation than the one previously described in mice. Finally, we observed that the HVEM/BTLA ratio is significantly lower in Tregs from lupus patients compared to healthy controls, whereas *ex vivo* effector CD4^+^ T cells express higher BTLA levels. Our data suggest that an altered expression of BTLA and HVEM could be involved in an impaired regulation of autoreactive T cells in lupus. These results provide a better understanding of the BTLA involvement in lupus pathogenesis and confirm that BTLA should be considered as an interesting target for the development of new therapeutic strategies.

## Introduction

As cytotoxic T lymphocyte-associated protein 4 (CTLA-4) and programmed cell death 1 (PD1), B and T lymphocyte attenuator (BTLA) is a co-inhibitory receptor through which immune responses can be negatively regulated (1). BTLA, a member of the immunoglobulin superfamily, has been detected on various immune cells including B and T lymphocytes, macrophages, dendritic cells and natural killer cells (2) and interacts with herpesvirus-entry mediator (HVEM) which is also widely expressed on hematopoietic cells (3). Following HVEM ligation to BTLA, immunoreceptor tyrosine-based inhibition motifs (ITIM) located in BTLA cytoplasmic tail become phosphorylated, triggering the recruitment of SHP1 (and to a lower extend SHP2) (4, 5) and the dephosphorylation of both the TCR and CD28. Consequently, BTLA engagement through HVEM leads to diminished cell activation, cytokine production and proliferation of BTLA-expressing cells, particularly T cells (6).

In the absence of BTLA, mice display autoimmune features such as auto-antibody (autoAb) production and increased lymphocyte proliferation (1). BTLA deficient mice are more susceptible than wild type counterparts to the development of experimental autoimmune encephalomyelitis (1) and autoimmune like-hepatitis (7) and exhibit enhanced hapten-induced contact hypersensitivity (8). Moreover, BTLA deficiency makes mice resistant to peripheral T cell tolerance induction (9), whereas the administration of an agonistic anti-BTLA antibody prevents the development of graft *versus* host disease [GVHD; (10)] and prolongs cardiac allograft survival (11). Altogether, these studies highlight the key role of BTLA in the maintenance of peripheral tolerance in mouse models. However, BTLA expression and function in T cells in human diseases as well as its contribution in peripheral tolerance remains poorly documented.

Systemic lupus erythematosus (SLE) is a severe systemic autoimmune disease characterized by a loss of self-tolerance. The etiology of SLE is not fully defined, but genetic, hormonal and environmental factors, as well as various immunological abnormalities, are implicated. Dysregulation of various components of the immune system can be observed at different stages of SLE development, but hyperactivity of B cells, leading to excessive production of multiple autoAb and their deposit into targeted organs such as skin and kidney, plays a crucial role in the severity of lupus disease (12). In that context, we previously demonstrated that some pathogenic antinuclear autoAb are produced by plasma cells, which are localized into inflamed kidneys of lupus mice (13, 14). B cells, as plasma cell precursors and autoAb producers, are thus considered as a major pathogenic cell subset in SLE. However, the T-B crosstalk, leading to B cell differentiation into plasma cells, represents a central element in lupus pathogenesis, and as such, CD4^+^ T cells are key contributors of the altered immune response as well. Among the large variety of CD4^+^ T cell subsets described in the literature, follicular helper T cells (T_FH_) and regulatory T cells (Tregs) caught a lot of attention from researchers working on lupus. Indeed, T_FH_ orchestrate the molecular interactions that provide help to B cells in secondary lymphoid organs (SLO) and are defined as facilitators of Ab production. In contrast, Tregs negatively regulate the immune response. In accordance with their respective functions, several groups have evidenced altered proportion and/or function of these two CD4^+^ T cell subsets in SLE, T_FH_ (or T_FH_ subsets) being enhanced (15, 16) whereas Tregs display reduced frequency (17).

To date, most research has focused on BTLA expression on the surface of total CD4^+^ T cells, and data regarding BTLA expression in these two major T cell populations in humans are missing. In mice, BTLA is highly expressed by T_FH_ localized in the germinal centers (GC) (18). The number of GC B cells is increased in BTLA deficient mice and adoptive transfer of T_FH_ deficient for BTLA to wild type mice induces more antigen-specific IgG2a and IgG2b production (19), indicating that BTLA expressed by T_FH_ may control GC B cell development. Concerning Tregs, Tao and colleagues showed that BTLA is up-regulated on TCR-stimulated effector T cells (Teffs) but expressed at very low levels by Tregs, whereas HVEM is mainly expressed by Tregs upon T cell activation (20). Co-culture of Tregs and Teffs isolated from BTLA and HVEM deficient mice revealed that Tregs exert suppression *via* up-regulation of HVEM, which binds to BTLA expressed by Teffs (20). The authors proposed that low BTLA expression on Tregs, favors effector T cell suppression while preventing HVEM-BTLA interactions between Tregs. Interestingly, in the MRL/lpr lupus mouse-model, characterized by T_FH_ hyperactivity and Tregs dysfunction, BTLA deficiency leads to exacerbation of lupus symptoms and reduced survival, suggesting that the BTLA pathway plays important roles in controlling the disease development (21).

We recently demonstrated that, in SLE, BTLA signaling is unable to properly inhibit CD4^+^ T cell activation, due to an impaired recruitment of this co-receptor to the immunological synapse following T cell stimulation (22). In this study, we did not detect significant *ex vivo* variations of BTLA expression on total CD4^+^ T cells between lupus patients and healthy controls (HC); however, very interestingly, we observed that the enhancement of BTLA expression following *in vitro* T cell activation is significantly lower in SLE patients compared to HC.

Considering the critical role of T_FH_ in plasma cell generation on the one hand, and of Tregs in maintaining peripheral tolerance on the other hand, we aimed in the present work at delineating the expression pattern of the co-inhibitory receptor BTLA on these two CD4^+^ T cell subsets in steady state and lupus settings.

## Materials and Methods

### Patients and Controls

A total of 47 SLE patients (40 women and 7 men, aged from 18 to 82 years) attending at University Hospital (Strasbourg, France) and 34 age and sex-matched healthy controls (HC) were enrolled in this study. All patients met the American College of Rheumatology criteria for classification of SLE (23) and disease activity was assessed by SLEDAI. Routine measures were used to determine anti-nuclear Abs (ANAs, by indirect immunofluorescence with Hep-2 cells) and anti-dsDNA (screened by ELISA; Kallestad anti-DNA microplate EIA, Bio-rad Lab. Inc., CA, USA). To avoid the effect of immunosuppressive agents on BTLA expression, all patients who received prolonged and heavy suppressive agents (cyclophosphamide, mycophenolate mofetil, azathioprine) or biologics (belimumab or rituximab in the previous 12 months) were excluded from our study. Patients included in the study were untreated or treated with methotrexate, hydroxychloroquine, and/or dose of steroids ≤15mg prednisone equivalent a day. Characteristics of SLE patients are listed in **Table 1**.

**Table 1:** Clinical and biological characteristics of SLE patients.

	SLE patients (n = 47)
***Sex (F/M)***	40/7
***Age (years), median (range)***	46 (18–82)
***SLEDAI, median (range)***	4 (0–21)
*n (%)*	
In remission (SLEDAI = 0)	15 (32)
Low activity (SLEDAI 1-5)	16 (34)
Mild activity (SLEDAI 6-10)	10 (21)
High activity (SLEDAI 11-19)	3 (6)
Very high activity (SLEDAI ≥ 20)	3 (6)
Clinical manifestations*	
Rash	8
Arthritis	15
Pleurisy/Pericarditis	5
Nephritis	7
***Biological features***	
Anti-dsDNA**	22
Low complements	19
Proteinuria	10
***Hematological features***	
Anemia	11
Lymphopenia	10
Leucopenia	5
Thrombocytopenia	3
Hematuria	5
***Treatment, median (range)***	
None	6
CS <10mg/day	16
*Median (range)*	*5* (2,5–7)
CS ≥10 mg/day	8
*Median (range)*	*10* (10–15)
HCQ (mg/day)	26
*Median (range)*	*400* (200–600)
MTX (mg/week)	9
*Median (range)*	*20* (10–25)

F, female; M, male; SLEDAI, SLE disease activity index; CS, corticosteroids; HCQ, hydroxychloroquine; MTX, methotrexate.

*at the time of blood drawn **considered positive when the titer was 50≥IU/ml as measured by ELISA.

### Ethics Statement

All samples were obtained from volunteers attending the Rheumatology Clinic of Strasbourg University Hospitals and were collected during routine clinical (diagnostic/prognostic/therapeutic) procedures prescribed. The ethical approval is not required for these types of studies under French legislation if no additional procedures are performed, as it is the case in our study. All patients provided written informed consent prior to their participation in the study in accordance with the Declaration of Helsinki.

### PBMC, CD4^+^ T Cell and Treg Isolation, and Cell Culture

Peripheral blood mononuclear cells (PBMCs) from lupus patients and from volunteers and anonymous donors of *Etablissement Français du Sang* were isolated from heparinized venous blood by Ficoll density gradient centrifugation (GE Healthcare). CD4^+^ T cells from healthy donors were negatively selected using the MojoSort Human CD4 T Cell Isolation Kit (Biolegend) according to the manufacturer’s instructions. The purity of the CD4^+^ population was typically ≥ 90%. Isolated CD4^+^ T cells were incubated with the following conjugated mAbs: anti-CD4-allophycocyanin (APC) Violet-770 (clone REA 623, Miltenyi); anti-CD45RA-phycoerythrin (PE) Violet-615 (clone 562, Miltenyi); anti-CD25-APC (clone REA 570, Miltenyi), and naive CD4^+^ T cells and Treg subpopulations (aTregs and rTregs) were isolated by a FACSAria™ Fusion cell sorter (BD). Purified naive CD4^+^ T cells, aTregs and rTregs were cultured in complete medium (RPMI 1640 containing 10% FCS, 10 µg/ml gentamicin, and 10 mM HEPES) and plated at 5.10^5^ cells per ml at 37°C. Cells were stimulated with 5 µg/ml plate-bound anti-CD3 (clone OKT3, eBioscience) and with 5 µg/ml soluble anti-CD28 (clone CD28.2, BD Pharmingen). Samples were cultured for 4 hours or 48 hours, harvested, and used for RNA isolation or flow cytometric analysis respectively.

### Flow Cytometry Analysis

**Figure 1:**
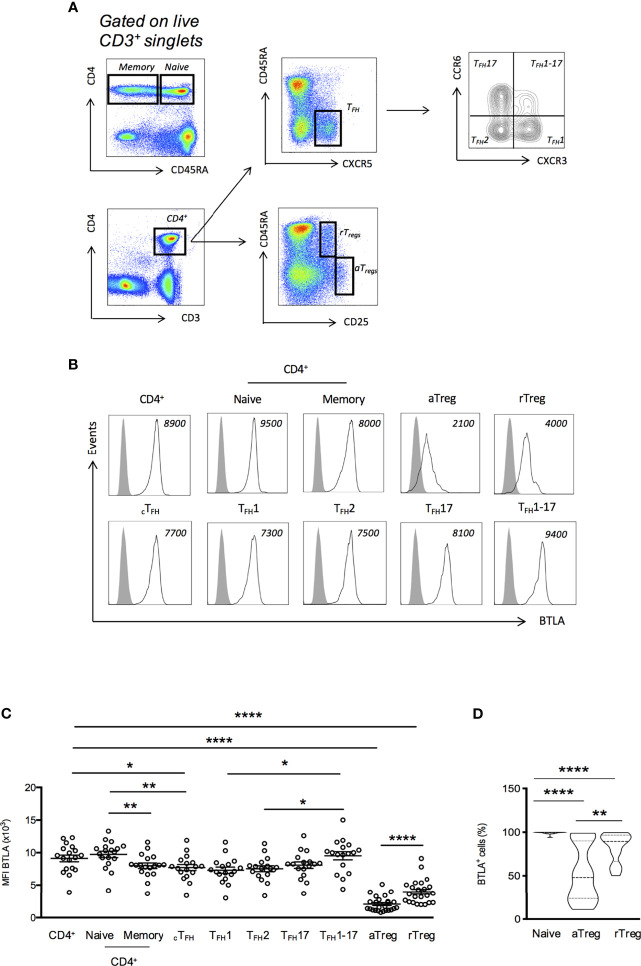
BTLA expression on T cell subsets from HC. **(A)** Flow cytometry gating strategy of T cell subsets defined by CD3, CD4, CD45RA, CXCR5, CCR6, CXCR3 and CD25. **(B)** Representative histograms of BTLA expression on T cell subsets. Data from a representative HC are shown as an example and MFI values are indicated. **(C)** BTLA expression (MFI) on T cell subsets in HC (n=17-34). **(D)** Frequency of BTLA expressing cells in naive CD4^+^ T cells, aTregs and rTregs in HC. Results are expressed as mean ± SEM and each dot represents one individual. Frequency values are displayed as mean (dashed lines) plus lower and upper quartiles (dotted lines). *p < 0.05; **p < 0.01; ****p < 0.0001, Mann-Whitney.

PBMCs, naive CD4^+^ T cells or Treg subpopulations were stained for 20 min at 4°C in staining buffer (2% FCS in PBS) with the following conjugated mAbs: anti-BTLA-PE (clone MIH26, Biolegend); anti-CD45RA-PE Violet-615 (clone 562, Miltenyi); anti-CXCR5-fluorescein (FITC) (clone REA103, Miltenyi); anti-HVEM-PE Cyanine-7 (PE-Cy7) (clone 122, Biolegend); anti-CD25-APC (clone REA 570, Miltenyi); anti-CD3-Alexa Fluor 700 (clone UCHT-1, BD Pharmingen); anti-CD4-APC Violet-770 (clone REA 623, Miltenyi); anti-CCR6-PE-Cy7 (clone 11A9, BD); anti-CXCR3-APC (clone REA232, Miltenyi). Dead cells were excluded using 4’,6-diamidino-2-phenylindole (DAPI) and single cells were discriminated from aggregates or doublets using SS-W *versus* SS-H and FS-W *versus* FS-H plots. Cell acquisition was performed using 10-color Flow Cytometer Gallios-Navios (Beckman Coulter). At least 1x10^6^ cells were analyzed using FlowJo 7.6.5 software (TreeStar) with the strategy depicted in **Figure 1** by using Fluorescence Minus One (FMO) controls to define gates.

### RNA Isolation, cDNA Synthesis, and Quantitative Real-Time PCR

To measure BTLA and HVEM mRNA expression, RNA was extracted from CD4^+^ T cells, aTreg or rTreg using an RNeasy Plus Micro Kit (Qiagen) according to the manufacturer’s instructions. First-strand cDNA was synthetized using the Maxima First Strand cDNA Synthesis kit for RT-qPCR (Thermo Scientific). The relative amount of each transcript was normalized against the mean expression of two housekeeping genes, namely glyceraldehyde 3-phosphate dehydrogenase (GAPDH) and beta-actin (ACTB). The qPCR primers and probes were purchased from ThermoFisher as predesigned TaqMan gene expression assays for the targeted genes as follows: *ACTB* (Hs99999903_m1); *GAPDH* (Hs99999905_m1); *BTLA* (Hs00699198_m1) and *HVEM* (Hs00187058_m1). All amplification reactions were performed in a total volume of 10 µl using a StepOnePlus real-time PCR system (Applied Biosystems) with TaqMan™ Gene Expression Master Mix (Applied Biosystems). The thermocycling conditions were: initial 10 min incubation at 95°C followed by 40 cycles of denaturation for 15 s at 95°C and annealing/extension for 1 min at 60°C. Data were collected with StepOne v2.1 software and the ΔΔCT method was used to calculate fold changes. Values of ΔCt and ΔΔCt are depicted in **Supplementary Table S1**.

### Statistics

Data were analyzed using GraphPad Prism version 6 or version 8 (GraphPad Software Inc). Differences between SLE patients and healthy individuals were determined with a Mann-Whitney test. Relationships between two variables were evaluated using Spearman’s correlation coefficient. Data are expressed as mean ± SEM, and differences were considered to be statistically significant at p<0.05 or less.

## Results

### *Ex Vivo* Expression of BTLA by CD4^+^ T Cell Subsets

In mice, BTLA is highly expressed by T_FH_ from SLO and is considered as a T_FH_ marker (18), whereas spleen Tregs express very low levels of BTLA (20). To date, the description of BTLA expression by human cells is mainly restricted to total CD4^+^ and CD8^+^ T cells and there are only few available data regarding circulating T_FH_ (cT_FH_) and Tregs. Therefore, we examined the expression of BTLA *ex vivo* in PBMCs isolated from HC by using a multicolor staining allowing to identify cT_FH_ (CD3^+^CD4^+^CD45RA^-^CXCR5^+^) and T_FH_ subsets (T_FH_1; CXCR3^+^CCR6^-^, T_FH_2; CXCR3^-^CCR6^-^, T_FH_17; CXCR3^-^CCR6^+^, and T_FH_1_-_17; CXCR3^+^CCR6^+^), as well as the two main Tregs subsets, as defined by Miayra and colleagues (24) i.e. activated Tregs (aTregs; CD45RA^-^CD25^hi^) and resting Tregs (rTregs; CD45RA^+^CD25^+^) (**Figure 1A**). In HC, BTLA is more highly expressed on naive CD4^+^ T cells than on memory CD4^+^ T cells (MFI 9738 ± 490 *vs* 8003 ± 431, p<0.01; **Figures 1B, C**). In cT_FH_ cells, the expression of BTLA was found to be diminished compared to naive CD4^+^ T cells (MFI 7722 ± 471 *vs* 9738 ± 490, p<0.01; **Figures 1B, C**). Moreover, among the four cT_FH_ subsets (T_FH_1, T_FH_2, T_FH_17 and T_FH_1_-_17), theT_FH_1-17 subset is the one that expresses the highest BTLA level (MFI 9533 ± 619 for T_FH_1-17 and 7722 ± 471 for T_FH_1, p<0.05). Concerning Tregs, our results revealed that, as in mice, BTLA expression is low on both Treg subsets from HC, the lowest BTLA expression being observed on the aTreg subset (MFI 2064 ± 228 for total aTregs and 3971 ± 366 for total rTregs, p<0.0001 compared to total CD4^+^ T cells; **Figures 1B, C**). The weak BTLA expression by Tregs is accompanied with a decreased frequency of BTLA^+^ expressing cells. Indeed, whereas BTLA is expressed on the majority of circulating CD4^+^ T cells [**Figure 1D**; median of 99% (range 89-100)], only about 50% of aTregs were found to express BTLA [48% (11-98)].

### Increased Expression of BTLA on Lupus aTregs

**Figure 2:**
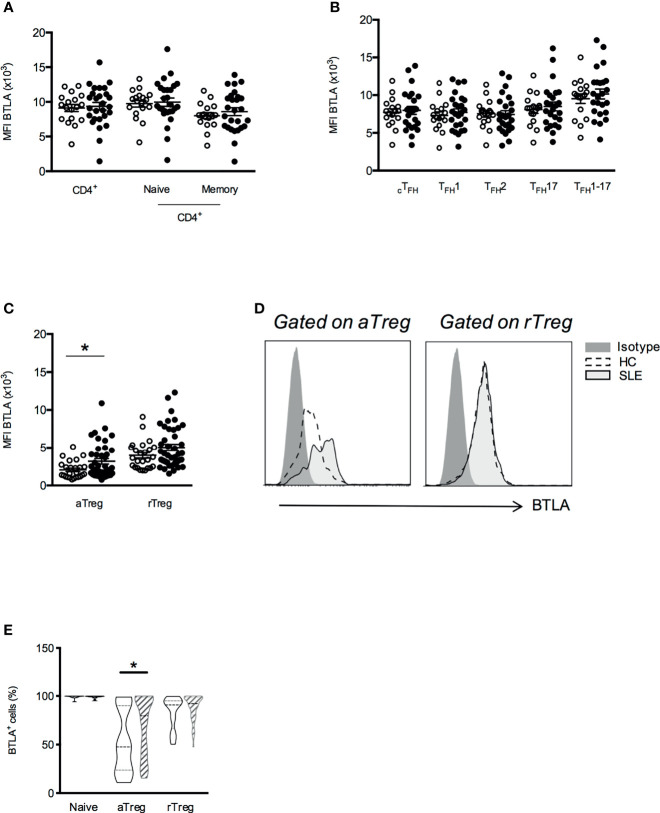
BTLA expression is enhanced on aTregs from SLE patients compared to HC. Comparison of BTLA expression (MFI) on CD4^+^ T cell **(A)**, cT_FH_ **(B)** and Treg subsets **(C)** in HC (white dots, n=17-34) and SLE patients (black dots, n=25-47). **(D)** Representative histograms of BTLA staining in aTregs and rTregs from one HC and one lupus patient. **(E)** Comparison of BTLA expressing cell subset frequencies between HC (empty boxes) and lupus patients (hatched boxes). Frequency values are displayed as mean (dashed lines) plus lower and upper quartiles (dotted lines). Results are expressed as mean ± SEM and each dot represents one individual. *p < 0.05, Mann-Whitney.

We next evaluated BTLA expression by CD4^+^ T cells isolated from 25-47 SLE patients (**Table 1**) compared to 17-34 age- and sex-matched healthy individuals. We found that BTLA expression on cT_FH_, T_FH_ subsets and naive and memory CD4^+^ T cells did not substantially differ between SLE patients and HC (**Figures 2A, B**). However, even if aTregs from lupus patients still express lower levels of BTLA than other lupus CD4^+^ T cells (MFI 3188 ± 357 *vs* 9358 ± 559 in total CD4^+^ T cells, p<0.0001), we noticed a significant increase of BTLA expression on lupus aTregs compared to HC aTregs (MFI 2064 ± 228 *vs* 3188 ± 357, p<0.05; **Figures 2C, D**) as well as an enhancement of the frequency of BTLA-expressing aTregs in lupus patients [median of 79% (range 15-100), **Figure 2E**]. This was not observed for rTregs, indicating that there is no systemic defect in the regulation of BTLA expression but rather a specific alteration in aTregs.

### Association of Enhanced BTLA Expression on aTregs and Clinical Parameters

**Figure 3:**
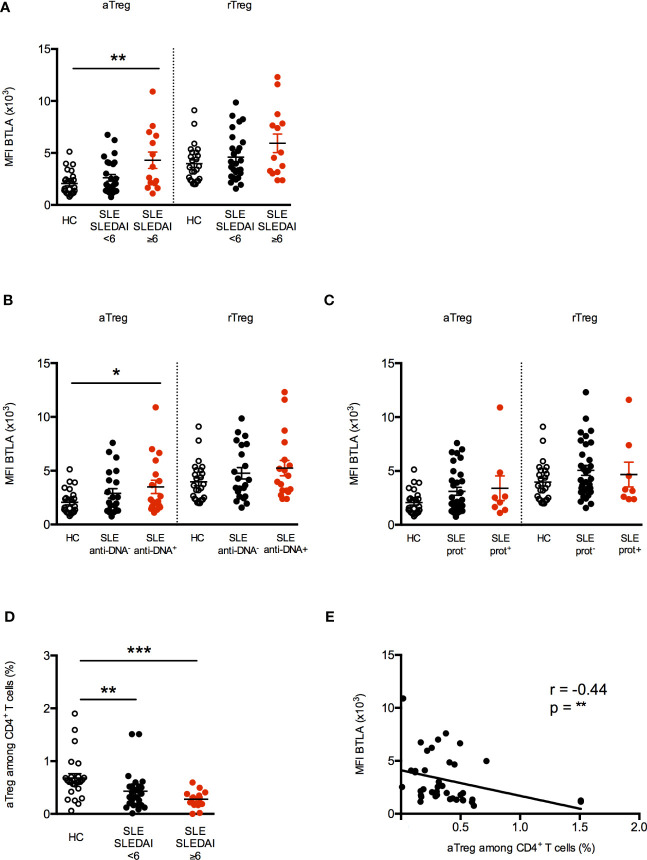
Increased BTLA expression correlates with decreased aTregs frequency in active SLE patients. **(A)** BTLA expression on aTregs and rTregs in HC (white dots, n=25), patients with inactive SLE or with low activity (SLEDAI <6; black dots, n=27) and patients with mild to severe SLE (SLEDAI≥6; red dots, n=14). **(B)** BTLA expression on aTregs and rTregs in HC (white dots, n=25), SLE patients without @DNA Abs (black dots, n=22) and SLE patients with @DNA Abs (red dots, n=18). **(C)** BTLA expression on aTregs and rTregs in HC (white dots, n=25), SLE patients without proteinuria (black dots, n=32) and SLE patients with proteinuria (red dots, n=8). **(D)** Frequency of aTregs among CD4^+^ T cells from HC (white dots, n=25), patients with inactive SLE or with low activity (SLEDAI <6; black dots, n=27) and patients with mild to severe SLE (SLEDAI≥6; red dots, n=14). Results are expressed as mean ± SEM and each dot represents one individual. **(E)** Correlation between BTLA expression (MFI) and the percentage of aTregs among CD4^+^ T cells (n=40). *p < 0.05; **p < 0.01; ***p < 0.001, Mann-Whitney test; r, Spearman correlation coefficient.

To investigate the clinical significance of higher BTLA expression on lupus Tregs, we then analyzed BTLA levels (MFI) in relation to disease activity assessed by the SLEDAI score. When patients were classified according to their disease activity status [inactive SLE to low SLE activity (SLEDAI<6) *versus* mild to severe SLE (SLEDAI≥6)], we found that BTLA expression was statistically higher on aTregs from patients with mild/severe SLE (**Figure 3A**; p<0.01). Moreover, the enhancement of BTLA expression on aTregs is significantly associated to the presence of circulating anti-dsDNA autoAbs, which are characteristic for lupus (MFI 3499 ± 620 in the group of patients with anti-dsDNA autoAbs *vs* MFI 2064 ± 228 in HC, p<0.05; **Figure 3B**). Interestingly, we did not observe any association between disease activity or the presence of anti-dsDNA and the level of BTLA expression in rTregs, which did not display increased BTLA expression in lupus patients compared to HC (**Figures 3A, B**). Finally, BTLA expression is not significantly higher on aTregs from patients harboring proteinuria compared to lupus patients with no proteinuria; however, the number of proteinuria positive patients in our cohort is low (n=8, **Figure 3C**). Importantly, higher BTLA expression on lupus aTreg was not related to the age or to the treatments received by lupus patients (**Supplementary Figures S1A, B**).

As previously described by Miyara and colleagues (24), the aTreg percentage was significantly diminished in lupus patients particularly those displaying an active disease (SLEDAI≥6; 0.28% ± 0.04 *vs* 0.67% ± 0.08 in HC, p<0.001, **Figure 3D**). Very interestingly, we observed that the expression level of BTLA on aTregs inversely correlated with their frequency in PBMCs from lupus patients (**Figure 3E**, p<0.01). No significant correlation was found between BTLA expression and rTreg percentages (**Supplementary Figure S2A**), the frequency of the latter being not altered in our lupus cohort (**Supplementary Figure S2B**). Our results suggest that the higher BTLA expression on the surface of lupus aTregs may account for the reduced frequency of this Treg subset in lupus patients.

### Dynamics of BTLA and HVEM Expression on Treg Subsets Following Activation

In mice, it was shown that Tregs can exert suppression *via* up-regulation of HVEM on their surface upon activation and subsequent binding to BTLA, which is highly expressed by activated Teffs (20). In this work, the authors suggested that low BTLA expression on Tregs favors Teff suppression by preventing Treg HVEM-Treg BTLA interactions in *cis*. To date, there is no data concerning the dynamics of BTLA and HVEM expression by Tregs in humans following activation.

**Figure 4:**
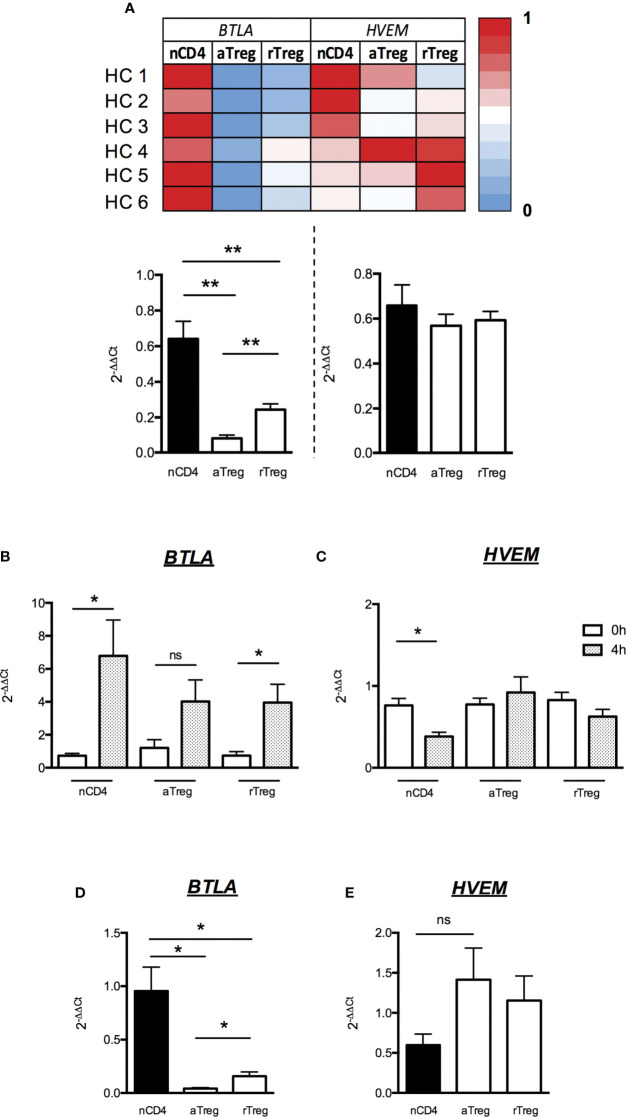
Low BTLA mRNA expression and maintained HVEM mRNA expression by Tregs after activation. **(A)** Levels of BTLA and HVEM transcripts were assessed by RT-qPCR on FACS-sorted naive CD4^+^ T cells (nCD4), aTregs and rTregs. Heatmap shows qPCR values of BTLA and HVEM transcripts normalized to GAPDH and ACTB. Results show average qPCR values from 6 HC and are expressed as fold change (2^-ΔΔCt^) compared to nCD4^+^ T cells. **(B, C)** BTLA and HVEM mRNA expression by naive CD4^+^ T cells, aTregs and rTregs from HC (n=4) following activation. Cells were stimulated with anti-CD3 (5µg/ml) and anti-CD28 (5µg/ml) Abs for 4 hours and harvested for analysis of BTLA and HVEM mRNA expression by RT-qPCR. Results show average qPCR values at t=4h and are expressed as fold change (2^-ΔΔCt^) compared to qPCR values at t=0. **(D, E)** Comparison of BTLA **(D)** and HVEM **(E)** mRNA expression between nCD4^+^ T cells, aTregs and rTregs from HCs (n=4) after 4 hours of stimulation (as described above). Results show fold changes (2^-ΔΔCt^) of qPCR values compared to nCD4^+^ T cells (n=4). Results are expressed as mean ± SEM. *p < 0.05; **p < 0.01, Mann-Whitney. ns, non significative.

We FACS-sorted aTreg and rTreg subsets as well as naive CD4^+^ T cells and performed quantitative real-time PCR in order to assess the expression of both BTLA and HVEM at the transcriptional level. Similarly, to what we found regarding protein expression by FACS, levels of BTLA-encoding mRNA were detectable but very low in both Treg subsets compared to naive CD4^+^ T cells (fold changes 0.08 ± 0.02 and 0.24 ± 0.03 for aTreg and rTreg respectively compared to naive CD4^+^ T cells, p<0.01, **Figure 4A**). On the contrary, all CD4^+^ T cell subsets express similar HVEM-encoding mRNA levels (**Figure 4A**). We then stimulated FACS-sorted CD4^+^ T cell subsets *in vitro* with anti-CD3/CD28 Abs and analyzed BTLA and HVEM mRNA and protein levels following 4h or 48h of stimulation respectively. As it was previously demonstrated in mice, TCR activation led to BTLA mRNA upregulation by naive CD4^+^ T cells (fold change 6.8 ± 2.2, p<0.05; **Figure 4B**) and to a lower extent by Tregs (fold change 3.9 ± 1.1 in rTregs, p<0.05 and 4 ± 1.3 in a Tregs, p=0.06; **Figure 4B**). In contrast, HVEM was down regulated in CD4^+^ T cells upon 4h of stimulation (fold change 0.4 ± 0.1, p<0.05; **Figure 4C**), whereas it remains unchanged in Tregs. However, the mRNA level of BTLA following 4h of stimulation was still significantly lower on both aTregs and rTregs (p<0.05; **Figure 4D**) whereas HVEM mRNA tend to be enhanced compared to naive activated CD4^+^ T cells (**Figure 4E**).

**Figure 5:**
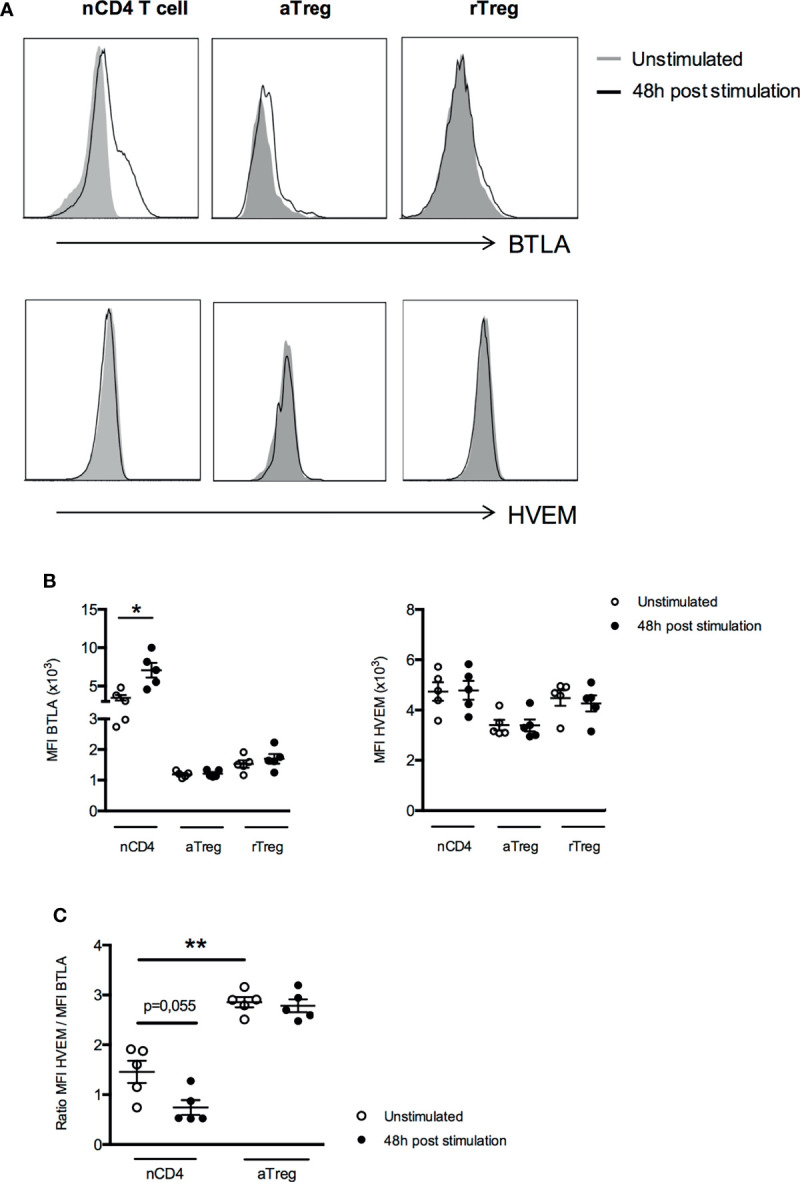
Expression of BTLA and HVEM proteins by Treg subsets do not change after activation. **(A)** Representative histograms of BTLA and HVEM expression on unstimulated naive CD4^+^ T cells (nCD4), aTregs and rTregs or following 48h of stimulation. Data from a representative experiment are shown as an example. **(B)** BTLA and HVEM expression (MFI) after 48 hours of stimulation (anti-CD3 5µg/ml and anti-CD28 5µg/ml, n=5). **(C)** Comparison of the ratio of HVEM and BTLA expression in naive CD4^+^ T cells and aTregs in the absence of stimulation (white dots, n=5) and following 48h of TCR stimulation (black dots, n=5). Results are expressed as mean ± SEM and each dot represents one individual. *p < 0.05; **p < 0.01, Mann-Whitney test.

At the protein level, we found that two days of activation led to a significant increase of BTLA expression at the surface of naive CD4^+^ T cells (MFI 7065 ± 958 *vs* 3475 ± 377, p<0.05) but not on aTregs and rTregs whereas HVEM protein expression remains unchanged in all CD4^+^ T cell subsets (**Figures 5A, B**). As HVEM and BTLA are co-expressed by the same cell, we calculated the ratio of HVEM and BTLA protein levels to evaluate their concomitant expression and their respective dynamics upon T cell stimulation. We observed a two-fold decrease of the HVEM/BTLA ratio in naive CD4^+^ T cells but not in aTregs (1.5 ± 0.2 *vs* 0.7 ± 0.1 in naive CD4^+^ T cells, p=0.055 and 2.9 ± 0.1 *vs* 2.8 ± 0.1 in aTregs, **Figure 5C**) following activation. In summary, our results show that although TCR stimulation led to its upregulation, BTLA expression by Tregs remains low compared to Teffs. On the contrary, HVEM mRNA expression (but not HVEM protein) is down regulated by activated CD4^+^ T cells but not by Tregs. Altogether, our results demonstrate for the first time, a similar dynamic of BTLA and HVEM expression pattern on Tregs and CD4^+^ T cells between human and mice following activation, suggesting that BTLA/HVEM-mediated suppression mechanisms previously described in the mouse-model may take place in the same way in humans.

### Decreased HVEM/BTLA Ratio in aTregs and Higher BTLA Expression by Effector CD4^+^ T Cells in Lupus Settings

**Figure 6:**
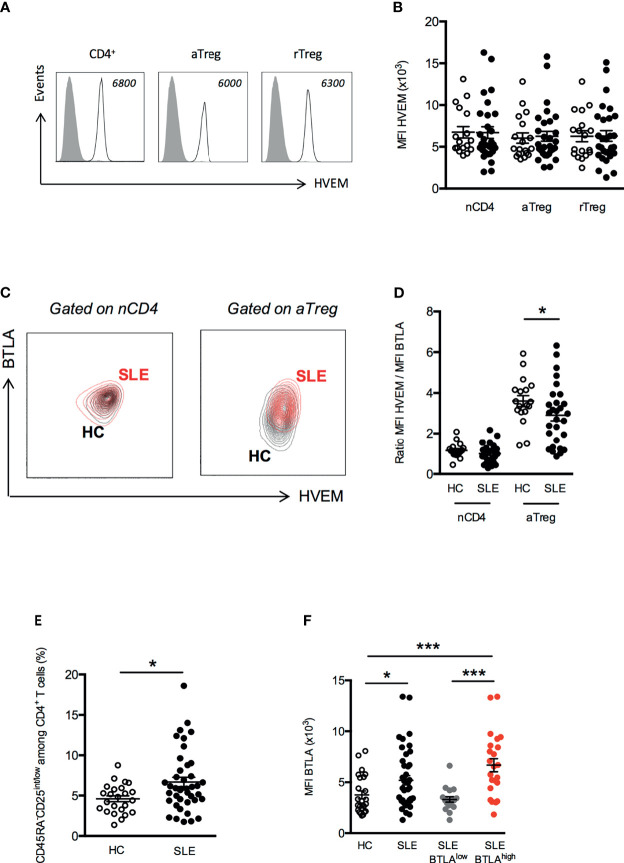
Disturbed HVEM/BTLA ratio on aTregs from SLE patients. **(A)** Representative histograms of HVEM expression on T cell subsets. Data from a representative HC are shown as an example and MFI values are indicated. **(B)** Comparison of HVEM expression (MFI) on naive CD4^+^ T cells (nCD4), aTregs and rTreg in HC (white dots, n=18) and SLE patients (black dots, n=29). **(C)** Representative dot plot of BTLA and HVEM expression on naive CD4^+^ T cells and aTregs from a HC (in black) and a SLE patient (in red). **(D)** Comparison of the ratio of HVEM and BTLA expression on naive CD4^+^ T cells and aTregs in HC (white dots, n=18) and SLE patients (black dots, n=29). **(E)** Frequency of CD45RA^-^CD25^int/low^ CD4^+^ effector T cells among CD4^+^ T cells from HC (white dots, n=24) and SLE patients (black dots, n=41). **(F)** BTLA expression on CD45RA^-^CD25^int/low^ CD4^+^ effector T cells in HC (white dots, n=24), SLE patients (black dots, n=41), SLE patients with low BTLA expression by aTregs (grey dots, n=18) and SLE patients with high BTLA expression by aTregs (red dots, n=23). Results are expressed as mean ± SEM and each dot represents one individual. *p < 0.05; ***p < 0.001, Mann-Whitney.

Our phenotypic analysis revealed a higher *ex vivo* BTLA expression on aTregs from lupus patients than HC. As HVEM plays a pivotal role in Treg-mediated suppression in mice, we compared the *ex vivo* expression of HVEM on peripheral CD4^+^ T cells and Tregs between HC and lupus patients. On the contrary to BTLA, all CD4^+^ T cells (naive CD4^+^ T cells, aTregs and rTregs) express similar levels of HVEM on their surface in HC (**Figures 6A, B**). Moreover, we did not notice any variation of HVEM expression on CD4^+^ T cells or Tregs from lupus patients compared to HC (**Figure 6B**). Due to the higher BTLA expression by lupus aTregs, the overlay of the BTLA/HVEM dot plots of a HC and a lupus patient, revealed an upward shift in lupus aTregs compared to HC aTregs (**Figure 6C**) and consequently, the calculated ratio of HVEM and BTLA expression on aTregs, which is of 3.6 ± 0.3 in HC is significantly decreased in aTregs from lupus patients (2.9 ± 0.3, p<0.05; **Figure 6D**) but not in other populations, such as naive CD4^+^ T cells. To investigate whether enhanced BTLA expression by lupus aTregs may have biological significance in the disease pathogenesis, we further focused on pathogenic effector T cells. We did not find any correlation between BTLA expression by lupus aTregs and circulating T_FH_ frequencies (not shown), but this result is consistent with the fact that there was no significant altered distribution of cT_FH_ (defined as CD45RA^-^CXCR5^+^ CD4^+^ T cells) in our lupus cohort compared to HC. On the contrary, and as previously described (24), the frequency of CD45RA^-^CD25^int/low^ defined by Miyara and colleagues as activated effector T cells **(** **Supplementary Figure S3**) with no suppressive activities but with high pro-inflammatory potential, is significantly higher in lupus patients (6.7 ± 0.6 in SLE patients *vs* 4.6 ± 0.4 in HC, p<0.05 **Figure 6E**). These lupus effector T cells express higher levels of BTLA than activated effector T cells from HC (MFI 3762 ± 387 in HC *vs* 5199 ± 461, p<0.05, **Figure 6F**) and more interestingly, the enhanced expression of BTLA by effector T cells is only observed in lupus patients displaying high BTLA expression levels by aTregs (MFI 3310 ± 268 in lupus patients with low BTLA expression by aTregs *vs* 6678 ± 646 in lupus patients with high BTLA expression by aTregs, p<0.001, **Figure 6F**).

## Discussion

The co-inhibitory receptor BTLA is nowadays clearly considered as a critical regulator of T cell responses, however, data regarding its expression in peripheral blood CD4^+^ T cell subsets are still limited. In the present work, we performed an in-depth analysis of BTLA expression on CD4^+^ T cell subsets suspected to play a key role in lupus pathogenesis, either by promoting the Ab-response or by limiting lymphocyte activation, i.e. cT_FH_ cells and Tregs respectively. Similarly, to what was described in CD8^+^ T cells (25, 26), we noticed that circulating CD4^+^ T cell subsets with a memory phenotype (CD45RA^-^ cells) express lower levels of BTLA than naive CD4^+^ T cells. Although BTLA is considered as a T_FH_ marker in mice, we did not observe a higher BTLA expression by cT_FH_ compared to other CD4^+^ T cell subsets. However, this result is not surprising and is consistent with other typical GC T_FH_ markers such as PD1 and Bcl6, which are highly expressed by tonsil T_FH_ but not by cT_FH_. Concerning Tregs, our results highlight that as in mice, human Tregs express very low levels of BTLA.

In the present work, we analyzed the *ex vivo* expression of BTLA by Tregs, but more importantly, we investigated the dynamic expression of BTLA and its ligand HVEM by Tregs and Teffs following TCR stimulation. As described by Tao and colleagues in mice (20), we evidenced that human Teffs up-regulate BTLA upon activation, whereas BTLA expression by Tregs remains low. On the contrary, TCR stimulation led Teffs to down-regulate and Tregs to slightly enhance their respective HVEM expression, at least at the mRNA level. Indeed, we were not able to detect any enhancement of HVEM protein expression by Tregs in our culture conditions. This results could be explained by a too short time of stimulation (48h) as the increase of HVEM expression by Tregs was evidenced only following 72h of stimulation in mice (20). As the differential expression of BTLA and HVEM by Tregs was shown to play an important role for Treg-suppressive functions both *in vitro* and *in vivo* in the mouse-model (20), one can speculate that a closely related mechanism may account for Treg functionality in human beings.

In this study, we found that *ex vivo* expression of BTLA on CD4^+^ T cells (both naive and memory) and on cT_FH_ was comparable between lupus patients and healthy individuals. Contrary to what we could expect, T_FH_2 cells, which we previously described as enhanced in active lupus patients (15), do not express lower levels of BTLA than other T_FH_ subsets. Very interestingly, we observed an altered expression of BTLA on lupus Treg subsets. Indeed, BTLA expression was significantly increased on terminally differentiated and highly suppressive aTregs, but not on rTregs (Tregs in a quiescent state that can differentiate into aTregs upon stimulation), of lupus patients compared to HC. BTLA expression by lupus Tregs was recently explored by two groups. Oster et al. reported similar frequencies of BTLA expressing Tregs (CD25^hi^CD127^-^) in lupus patients compared to healthy individuals, however, levels of BTLA expression by Tregs were not assessed in this study (27). Murphy et al. recently described a lower expression of BTLA on Tregs (defined as CD25^hi^CD127^lo^CD4^+^ T cells) compared to Th1 and Th17 cells in lupus patients (28), but they did not observe any significant variation of BTLA expression on Tregs between HC and SLE patients. However, lupus patients (only 5) analyzed in this study displayed low SLEDAI scores between 4-6, and accordingly in our cohort, BTLA expression by aTregs is only significantly enhanced in patients having a SLEDAI score higher than 6.

**Figure 7:**
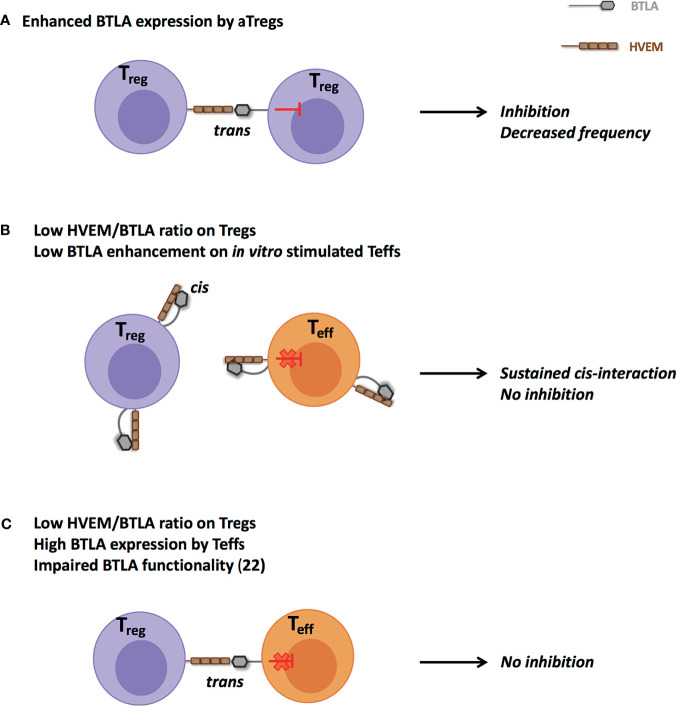
Proposed model for impaired HVEM-mediated suppression by Tregs in lupus. **(A)** Enhanced *ex vivo* BTLA expression by lupus aTregs could favor their own inhibition and participate to their reduced frequency. **(B)** Increased *ex vivo* BTLA expression by aTregs and defective BTLA enhancement in *in vitro* TCR-stimulated CD4^+^ T cells could sustain *cis*-interaction and thus limit *trans*-interaction between lupus Tregs and effector T cells. **(C)** Despite higher BTLA expression, altered BTLA signaling pathway in effector CD4^+^ T cells (22) could prevent their effective inhibition.

What are the potential consequences of such a higher BTLA expression by lupus aTregs? The most obvious answer is the BTLA-mediated inhibition of Treg cells. Our data support this hypothesis, as BTLA expression by lupus aTregs strongly correlates with decreased frequencies of aTregs in PBMCs from lupus patients and we propose a model in which HVEM-expressing Tregs mediate BTLA-expressing Tregs inhibition through *trans*-interaction (**Figure 7A**). Another outcome could be directly linked to Treg functionality. One limitation of the current study is that due to the very low frequency of aTregs in lupus patients (particularly those with a mild/severe disease), we were not able to collect enough cells to perform functional experiments that would allow to demonstrate that a higher BTLA expression impairs the suppressive ability of HVEM-expressing Tregs. As Treg-mediated suppression was described to be defective in SLE patients (29, 30), understanding whether defective BTLA expression could influence the function of lupus Tregs would be particularly interesting. In resting T cells, HVEM preferentially interacts with BTLA expressed on the same cell (31, 32) thus preventing the binding of other signaling molecules to HVEM. Indeed, HVEM is able to bind various ligands in addition to BTLA (33). The *trans*-interaction of HVEM with any of these ligands [BTLA but also LIGHT, CD160 and lymphotoxin α (34)], leads to the activation of the NF-κB pathway in HVEM-expressing cells. The HVEM-BTLA *cis*-interaction was proposed to play a role in maintaining T cell tolerance by impeding the establishment of *trans*-interaction and holding HVEM in an inactive state. Upon T cell activation, there is an inverse correlation between BTLA and HVEM expression [(35, 36) and the present work] allowing *trans*-interactions. Interestingly, we have previously shown that BTLA expression is significantly diminished in lupus CD4^+^ T cells compared to HC following *in vitro* TCR activation (22). In the present work, we evidenced that *ex vivo* lupus Tregs express higher levels of BTLA. We hypothesize that enhanced BTLA expression by lupus aTregs could sustain HVEM-BTLA *cis*-interactions and limit HVEM availability on Tregs. In parallel BTLA enhancement on stimulated CD4^+^ T cells is likely not sufficient enough to disrupt HVEM/BTLA *cis*-interactions on effector T cells and to allow HVEM^+^Treg binding in *trans* to BTLA^+^Teffs (**Figure 7B**). This proposed model is supported by the impaired suppressive functions of lupus Tregs *in vitro* as described by several groups. Surprisingly, and contrary to what was observed in *in vitro* stimulated T cells, we noticed that *ex vivo* CD4^+^ effector T cells from lupus patients express higher levels of BTLA than those from HC, suggesting that lupus Teffs may be efficiently suppressed *in vivo*. However, our previous study revealed that despite normal levels of BTLA expression, CD4^+^ T cells from lupus patients display an altered functionality of the BTLA signaling pathway (22), and may thus consequently be refractory to Treg-mediated suppression (**Figure 7C**). Altogether, our data suggest that the altered BTLA expression by lupus Tregs and effector T cells may contribute to decreased numbers of Tregs and potentially to their reduced-suppressive activity.

In the view of developing new therapeutic strategies for SLE, identifying molecular and/or cellular elements leading to enhanced BTLA expression on lupus aTregs is an open avenue. Very little is known about the factors that regulate BTLA expression but among them are microRNA (miRNA) that recently emerged as important regulators of immune responses. In mice, it was shown that miR155 targets the BTLA 3’UTR region and that knockdown of miR-155 in CD4^+^ T cells resulted in upregulation of BTLA expression (37). Interestingly, miR155 is highly expressed by Tregs and miR-155 deficient mice display significant decreased numbers of Tregs (38). In human autoimmune diseases, a decrease in the upregulation of miR155 in stimulated Tregs was evidenced in rheumatoid arthritis (39). Further studies are required to define whether miR155 (and/or others) effectively targets BTLA in human and whether a dysregulated miRNA expression by lupus aTregs may account for enhanced BTLA expression. BTLA expression could also be regulated thanks to direct interactions with cells expressing HVEM. Indeed, it was previously demonstrated that TCR ligation by HVEM-expressing cells led to downregulation of BTLA on antigen-specific T cells (26). In contrast, in the absence of HVEM, antigen-triggered BTLA downregulation was less pronounced and led to higher BTLA expression levels at late time points. Moreover, levels of soluble HVEM (sHVEM) were described to be enhanced in autoimmune or infectious contexts (40). Whether sHVEM directly influences BTLA expression is not known but we wondered whether there is a link between BTLA expression and sHVEM levels in our lupus cohort. However, we did not evidence any enhancement of sHVEM in lupus patients compared to HC (unpublished results), nor any correlation between sHVEM in lupus sera and the higher level of BTLA expression by lupus a Tregs.

In conclusion, this study deepens our knowledge regarding the expression of BTLA in CD4^+^ T cells, not only in SLE but also in healthy settings. Indeed, we provide evidences of BTLA and HVEM expression and their respective dynamics following activation on human Tregs, which has never been described before. We evidenced a higher expression of the co-inhibitory receptor BTLA in lupus aTregs, and our results support the hypothesis of a link between this observation and the diminution of aTreg frequency in lupus settings. An extensive knowledge of BTLA expression on all immune cell subsets involved in lupus pathogenesis is absolutely required to envisage targeting this molecule in the context of new therapeutic strategies. Our results contribute to this progress and may open the door to the development of new drugs and therapeutic approaches for SLE patients in the future.

## Data Availability Statement

The original contributions presented in the study are included in the article/**Supplementary Material**. Further inquiries can be directed to the corresponding author.

## Ethics Statement

Ethical review and approval was not required for the study on human participants in accordance with the local legislation and institutional requirements. The patients/participants provided their written informed consent to participate in this study.

## Author Contributions

FM designed the study. LA performed the experiments and analyzed the data. LA and FM wrote the manuscript. RF and J-EG participated in sample collection and clinical analysis and reviewed the article. MS and HD participated to discussions and reviewed the article. All authors contributed to the article and approved the submitted version.

## Funding

This work was supported by the French Centre National de la Recherche Scientifique (CNRS), the Fondation Arthritis-Courtin (grant to FM) and the French “Ministère de l’Enseignement et de la Recherche” (fellowship to LA).

## Conflict of Interest

The authors declare that the research was conducted in the absence of any commercial or financial relationships that could be construed as a potential conflict of interest.

## Publisher’s Note

All claims expressed in this article are solely those of the authors and do not necessarily represent those of their affiliated organizations, or those of the publisher, the editors and the reviewers. Any product that may be evaluated in this article, or claim that may be made by its manufacturer, is not guaranteed or endorsed by the publisher.
