# Supplementary material for: High BTLA Expression Likely Contributes to Contraction of the Regulatory T Cell Subset in Lupus Disease
Source: Front Immunol. 2021 Nov 25;12:767099. doi: 10.3389/fimmu.2021.767099 (PMC8656397; doi:10.3389/fimmu.2021.767099)
Supplement: Supplementary file 1 [file DataSheet_1.pdf]

A

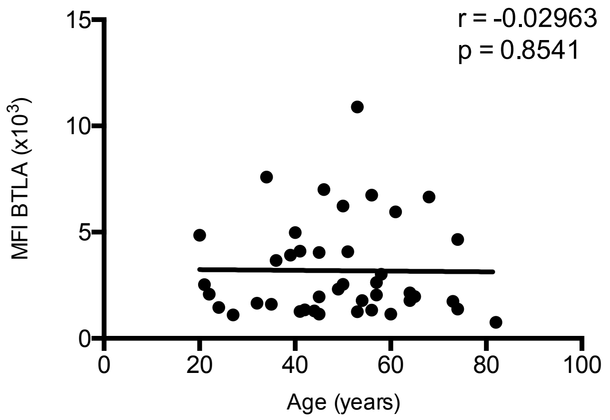

B

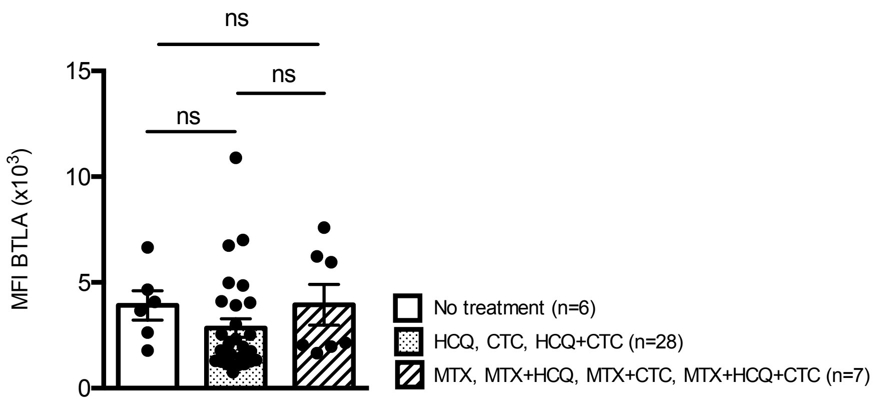

Figure S1. BTLA expression on aTregs from SLE patients does not correlate with treatments or age. **(A)** Correlation between BTLA expression (MFI) on aTregs from SLE patients and age. **(B)** BTLA expression (MFI) on aTregs from SLE patients according to treatments. Results are expressed as mean ± SEM and each dot represents one individual. r, Spearman correlation coefficient; HCQ, hydroxychloroquine; CTC, corticosteroids; MTX, methotrexate.

Supplementary S1

A

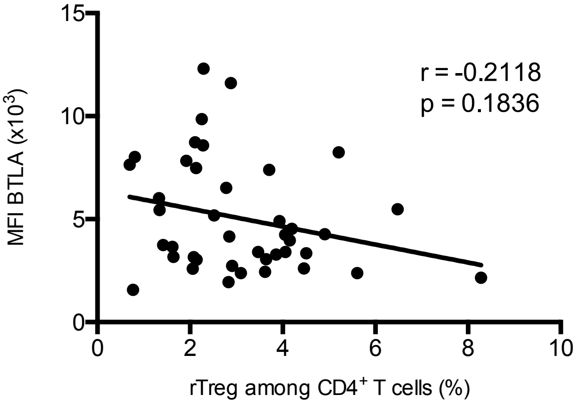

B

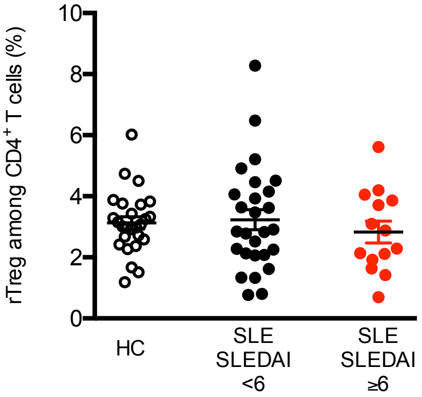

Figure S2. BTLA expression on lupus rTregs does not correlate with rTregs frequency. **(A)** Correlation between BTLA expression (MFI) on rTregs from SLE patients and the frequency of rTregs. **(B)** Frequency of rTregs among $CD4^+$ T cells from HC (white dots, n=26) patients with inactive SLE or with low activity (SLEDAI<6; black dots, n=27) and patients with mild to severe SLE (SLEDAI≥6 ; red dots, n=14). Results are expressed as mean ± SEM and each dot represents one individual. r, Spearman correlation coefficient.

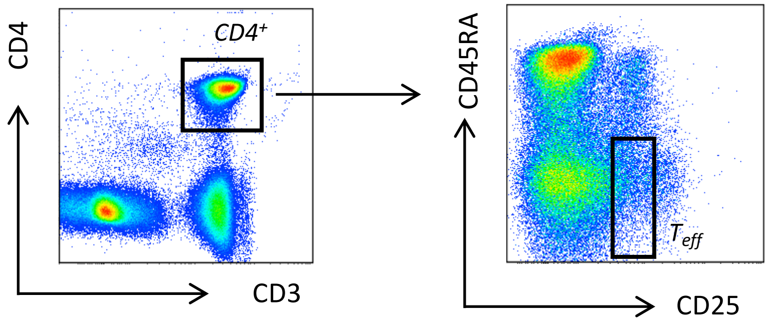

Figure S3. Flow cytometry gating strategy of effector T cells (CD45RA$^-$CD25$^{int/low}$) defined by CD3, CD4, CD45RA and CD25.

A

	BTLA 0h			HVEM 0h			BTLA 4h			HVEM 4h		
	**nCD4**	**aTreg**	**rTreg**	**nCD4**	**aTreg**	**rTreg**	**nCD4**	**aTreg**	**rTreg**	**nCD4**	**aTreg**	**rTreg**
S1	5,664	8,565	7,532	2,264	2,568	3,194	3,905	8,317	6,755	3,789	4,584	4,928
S2	6,247	10,365	8,684	2,507	3,624	3,471	4,345	9,398	7,151	4,744	3,940	4,663
S3	6,089	10,423	7,967	2,822	3,724	3,578	3,267	8,523	6,939	5,129	3,896	4,167
S4	7,265	11,107	9,668	3,500	4,251	4,355	4,843	8,822	6,748	4,881	4,907	4,672
S5	6,914	11,413	9,529	3,272	4,225	3,648						
S6	6,187	8,995	7,591	3,239	4,246	3,921						

B

	BTLA 0h			HVEM 0h			BTLA 4h			HVEM 4h		
	**nCD4**	**aTreg**	**rTreg**	**nCD4**	**aTreg**	**rTreg**	**nCD4**	**aTreg**	**rTreg**	**nCD4**	**aTreg**	**rTreg**
S1	0	2,900	1,868	0	0,304	0,929	0	4,412	2,850	0	0,794	1,138
S2	0,583	4,117	2,437	0,243	1,116	0,963	0,440	5,052	2,805	0,954	-0,803	-0,080
S3	0,424	4,334	1,877	0,558	0,901	0,755	-0,637	5,256	3,672	1,339	-1,232	-0,962
S4	1,601	3,841	2,403	1,236	0,751	0,852	0,938	3,978	1,904	1,091	0,026	-0,208
S5	1,249	4,499	2,615	1,008	0,953	0,376						
S6	0,523	2,807	1,403	0,975	1,007	0,682						

Supplemental Table 1. ΔCt (A) and ΔΔCt (B) values for BTLA and HVEM. S, Sample.
